# Rapid MR assessment of left ventricular systolic function early after acute myocardial infarction using single breath-hold cine imaging with temporal parallel acquisition technique (TPAT) and 4D guide-point modeling

**DOI:** 10.1186/1532-429X-11-S1-P180

**Published:** 2009-01-28

**Authors:** Holger C Eberle, Christoph J Jensen, Kai Nassenstein, Thomas Schlosser, Georg V Sabin, Christoph K Naber, Oliver Bruder

**Affiliations:** 1 Department of Cardiology and Angiology, Elisabeth Hospital Essen, Essen, Germany; 2 Department of Diagnostic and Interventional Radiology and Neuroradiology, University Hospital Essen, University of Duisburg-Essen, Essen, Germany

**Keywords:** Leave Ventricular Ejection Fraction, Cardiac Magnetic Resonance, Acute Myocardial Infarction, Delayed Enhancement, Leave Ventricular Ejection Fraction Measurement

## Introduction

Systolic left ventricular ejection fraction (LVEF) is a strong prognosticator after acute myocardial infarction (AMI). Currently, section summation of contiguous cardiac magnetic resonance (CMR) short axis steady-state free precession (SSFP) images is the reference standard for measuring LVEF.

However, the use of standard CMR LVEF measurement in the early postinfarction phase is limited by the time need of image acquisition in multiple breath-holds, which is frequently not well tolerated by critically ill patients. Additional time is needed for calculating LVEF by summation of discs by semi-automated post-processing tools.

Cine imaging accelerated by Temporal Parallel Acquisition Technique (TPAT) allows for the acquisition of multiple cine images in a single breath-hold. LVEF calculation from TPAT images in short an long axis orientation can be performed by a novel software tool applying four dimensional guide point modeling ventricular function analysis (4DVF). As 4DFV is based on geometric assumptions of a normal heart, it is unclear whether 4DFV is applicable to patients with AMI.

## Purpose

To compare 4D guide-point modeling LVEF analysis of TPAT images with standard LVEF analysis in patients with AMI.

## Methods

27 consecutive patients (18 male, mean age 60 ± 13 years) underwent CMR on a 1.5 Tesla MRI scanner (Avanto™, Siemens Medical Solutions, Germany) within 1 to 15 (mean 4) days after AMI.

The CMR protocol included SSFP cine imaging (TrueFISP, TR 3 ms, TE 1.5 ms, FA 60°, sl 3 mm) in 7–16 (mean 13) breath-holds and inversion recovery (IR) delayed enhancement imaging following gadolinium contrast administration.

Contiguous short axis cine images were analyzed by standard left ventricular function (LVF) analysis and the disc summation method (Argus™, Siemens Medical Solutions, Germany).

Additionally, two long axis and four short axis cine images were acquired in a single breath-hold using a TPAT accelerated SSFP sequence (TR 2,7 ms, TE 1,12 ms, FA 78°, sl 6 mm). The resulting cine images were used for 4-dimensional guide point modeling LVF analysis (Argus 4D™, Siemens Medical Solutions, Germany).

Both LVEF calculation methods were performed by two independent observers, and the duration of analysis was recorded.

## Results

**Figure 1:**
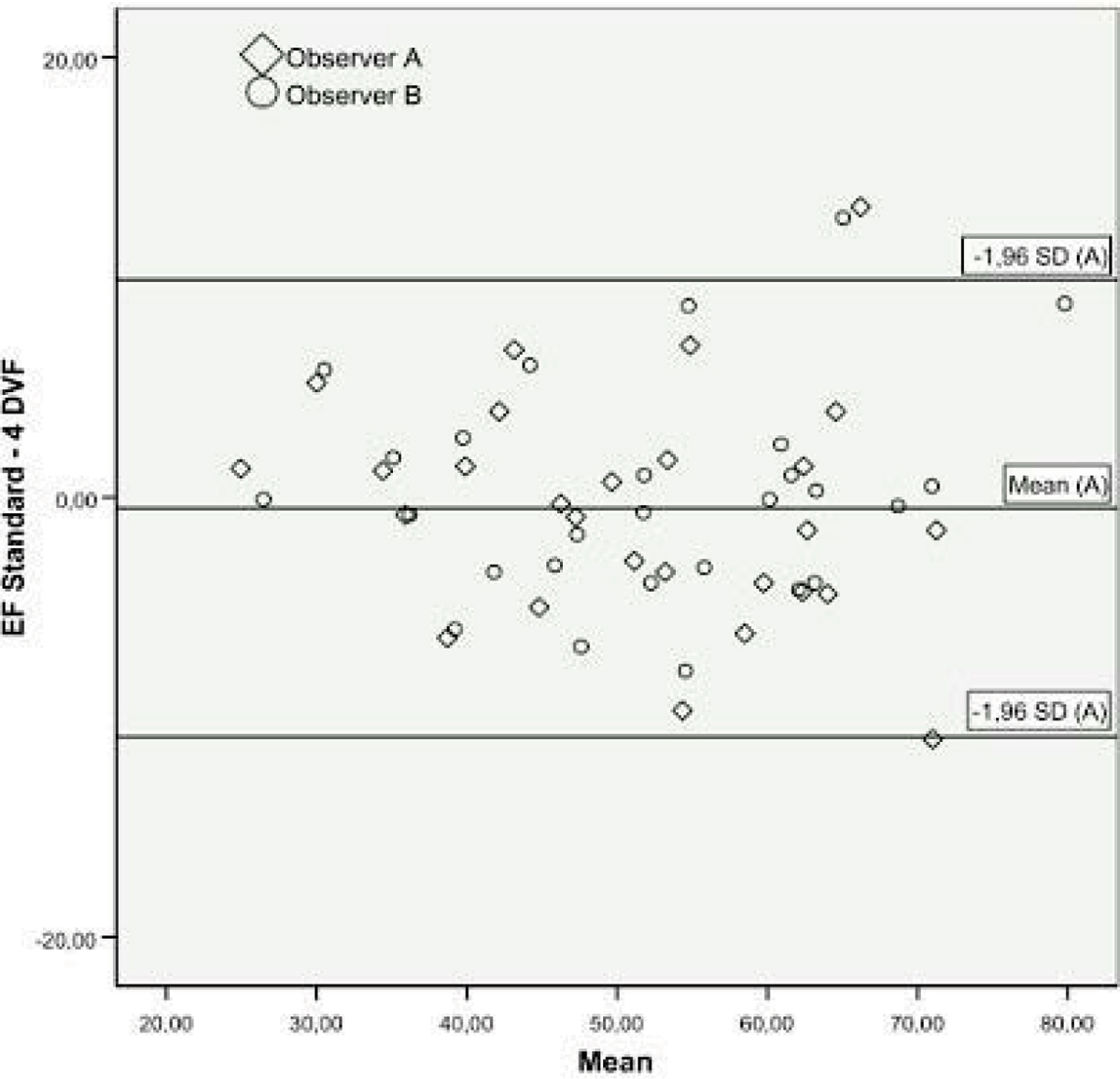
**Bland-Altman plots shows good agreement of both methods for calculating left ventricular ejection fraction (LVEF) for both observers**. Mean ± 1.96 * standard deviation is indicated for observer A.

Mean infarct size was 17 ± 12% of left ventricular mass. 6 ± 3 (range 1 to 12) LV myocardial segments were affected by wall motion abnormalities. 11 patients had anterior, 16 inferior wall myocardial infarction. A strong correlation of EF values from 4DVF with standard LVF analysis was observed (r = 0.92, p < 0.01). The results for observer A are shown in Table 1. Interobserver variability was low for both methods, and there were no statistically significant differences for LVEF (4DVF: 1.3 ± 4.4 %, p = 0.15 vs. standard LVF: -0.5 ± 2.8%, p 0.36) and LV Volumes (Table 2). For a Bland-Altman plot see figure 1. LVF Analysis was faster with the 4DVF approach (159 ± 84 s) as compared to the standard Argus procedure (276 ± 107 s, p < 0.01).

**Table 1:** LV ejection fraction, volumes and analysis time (Observer A).

	Standard LVF	4DVF	Mean Paired Difference
Ejection Fraction (%)	51.1 ± 12.3	51,6 ± 13.3	-0.54 ± 5.3
LV Enddiastolic Volume (ml)	165.7 ± 63.0	157.5 ± 64.4	8.2 ± 17.4
LV Endsystolic Volume (ml)	86.4 ± 50.1	81.8 ± 51.2	4.7 ± 12.2
LV myocardial mass (g)	160.4 ± 49	173.5 ± 54	-13.1 ± 44.9
Analysis Time (s)	305.7 ± 117.2	163.4 ± 79.4	142.1 ± 121.4*

Data are mean ± SD *p < 0.05.

**Table 2:** Interobserver variaion between stard LVF and 4DVR analysis.

	Standard LVF	4DVF
Ejection Fraction (%)	-1.3 ± 4.4	-0.5 ± 2.8
LV Enddiastolic Volume (ml)	3.3 ± 15.8	1.4 ± 11.0
LV Endsystolic Volume (ml)	3.3 ± 10.4	0.9 ± 11.1
LV Myocardial Mass (g)	4.6 ± 17.9	3.5 ± 32.5
Analysis Time (s)	59.2 ± 68.2*	8.7 ± 71.7

*p < 0.05 for difference.

## Conclusion

4DVF guide point modeling is a time saving alternative to standard LVF analysis. On the basis of images acquired in a single breath-hold, it yields results that are in good accordance with standard LVF analysis.

A protocol combining TPAT accelerated single breath-hold imaging and 4DVF image processing may be potentially useful for the rapid assessment of LV function in critically ill patients following acute myocardial infarction.

